# Supplementary material for: Inuit knowledge of Arctic Terns (Sterna paradisaea) and perspectives on declining abundance in southeastern Hudson Bay, Canada
Source: PLoS One. 2020 Nov 17;15(11):e0242193. doi: 10.1371/journal.pone.0242193 (PMC7671561; doi:10.1371/journal.pone.0242193)
Supplement: S1 File — (DOCX) [file pone.0242193.s001.docx]

**S1 File. Positionality of authors**

We would like to acknowledge co-author positionality, expertise and contribution to this study (Anwar and Viqar, 2017; Hoppers, 2009; Sultana, 2007). Manuscript authors included three southern Canada-based government researchers from ECCC (D.A. Henri [DAH], H.G. Gilchrist [HGG] and L.M. Martinez-Levasseur [LMML]), one Nunavik-based researcher affiliated with the Nunavik Marine Region Wildlife Board (F. Jean-Gagnon [FJG]), one southern Canada-based researcher from Acadia University (M.L. Mallory [MLM]), and one resident from Kuujjuaraapik (S. Weetaltuk [SW]), who was manager of the Local Nunavimmi Umajulivijiit Katujaqatigininga (LNUK) of Kuujjuaraapik and a board member with the Nunavik Marine Region Wildlife Board when this manuscript was submitted. SW was also interviewed as part of the project. All authors identify as non-Indigenous researchers, except for SW, who is an Inuk from Kuujjuaraapik. Co-authors contributed diverse and complementary expertise, including: knowledge of community culture, history and customs (SW); marine bird and Arctic Tern research in Nunavik and Arctic Canada (FJG, HGG, MLM); experience in transdisciplinary community-based environmental research and documentation of TEK/LEK in Nunavik and Arctic Canada (DAH, LMML). Two authors (FJG and SW) were directly involved in project activities taking place in Kuujjuaraapik. While SW was a long-term resident, FJG had never visited the community prior to this study.

**References**

Anwar, N.H., Viqar, S., 2017. Research assistants, reflexivity and the politics of fieldwork in urban Pakistan. Area 49, 114–121. https://doi.org/10.1111/area.12307

Hoppers, W., 2009. Participatory practices in policy-making: Negotiating democratic outcomes or manoeuvring for compliance? Int. J. Educ. Dev. 29, 250–259. https://doi.org/10.1016/j.ijedudev.2008.02.004

Sultana, F., 2007. Reflexivity, Positionality and Participatory Ethics: Negotiating Fieldwork Dilemmas in International Research. ACME An Int. E-Journal Crit. Geogr. 6, 374–385.
