# Supplementary material for: Inuit knowledge of Arctic Terns (Sterna paradisaea) and perspectives on declining abundance in southeastern Hudson Bay, Canada
Source: PLoS One. 2020 Nov 17;15(11):e0242193. doi: 10.1371/journal.pone.0242193 (PMC7671561; doi:10.1371/journal.pone.0242193)
Supplement: S2 File — (DOCX) [file pone.0242193.s002.docx]

**S2 File. Interview guide for semi-directed interviews with Inuit experts.**

Interviewees could skip or add topics/questions depending on their expertise. Questions could have been asked in any order, depending on the flow of the conversation. A topographic map was used during the interviews to record spatial observations.

**Section 1. Life history**

1. Where and when were you born?
2. How long have you lived in this community for?
3. What other communities have you lived in?
4. Did/do you hunt or accompany hunters on the land? If yes, where do you go, when and how often?
5. Did/do you fish or accompany people who fish? If yes, where would you go, when and how often?
6. Did/do you visit bird colonies?
7. If so, where did/do you go, when and how often?
8. If so, what did/do you do at the bird colonies?

**Section 2. Cultural significance and harvesting practices specific to Arctic Terns**

1. Are Arctic Terns important for you? If so, why?
2. Have you collected Arctic Tern eggs in the past? If so, when was the last time you collected Arctic Tern eggs?
3. What have you used Arctic Terns for throughout your lifetime (eating eggs, making tools)?
4. Do you think knowledge about Arctic Terns is changing among the younger generation? If so, why?

**Section 3. Range, distributional shifts and hotspot locations**

1. Where and when do you see Arctic Terns nowadays? (including colonies)
   1. Approximately how many terns do you see in these areas?
   2. What do terns do in these areas (nesting, feeding, migrating)?
2. Where and when did you used to see Arctic Terns in the past? (including colonies) (for each sighting, ask for more information about specific timescale)
   1. Approximately how many terns did you see in these areas?
   2. What do they do in these areas (nesting, feeding, migrating)?
3. Have Arctic Terns moved away from some areas?
   1. If so, when did this happen?
   2. If so, how do you explain this?
4. Nowadays do you see Arctic Terns in areas where they did not used to be in the past?
   1. If so, when did this happen?
   2. If so, how do you explain this?

**Section 4. Population trends and potential stressors**

1. Have you noticed any changes in Arctic Terns abundance (how many Arctic Terns you see)?
2. In your opinion, has the Arctic Tern population (how many Arctic Terns there are overall in your region based on your observations) increased, decreased or remained the same over your lifetime? Over the last 10 years? How would you explain that?
3. Have you ever seen any sick or dead Arctic Tern or other birds (that did not die from wounds caused by hunting)?
4. Have you seen Arctic Terns eating?
   1. Where and what do they eat?
   2. Have you noticed any changes in where/what they are eating?
5. What animals eat Arctic Terns and/or their eggs?
   1. Have you noticed any changes in how many Arctic Terns and/or tern eggs are eaten by these predators?
   2. If so, how do you explain this/these change(s)?
6. What makes a good habitat (place to live) for Arctic Terns in this region? Have you noticed any changes in Arctic Tern habitat?
7. Do you have any concerns about Arctic Terns?

**Section 5. Management actions**

Prior to asking the following questions, the interviewer presented a brief overview of the status of Arctic Terns populations from a science perspective.

1. Based on your experience and the information provided, what do you think should be done? (please choose one of the three following options)
   1. Take action to help Arctic Tern populations
   2. Let nature take its course (do nothing)
   3. No opinion
2. If ‘Option A’ selected, which of the following actions do you think should be taken? Please choose one or more of the following options (the interviewer presented additional information for each option, as needed):
   1. more protection of Arctic Tern habitat
   2. restrictions on egg picking
   3. more research (abundance surveys, contaminant studies, tracking studies, community-based studies)
   4. other
3. Have you ever heard other people in Nunavik express concerns about Arctic Terns?
4. Is there anything else you would like to add?
