# Supplementary material for: Inuit knowledge of Arctic Terns (Sterna paradisaea) and perspectives on declining abundance in southeastern Hudson Bay, Canada
Source: PLoS One. 2020 Nov 17;15(11):e0242193. doi: 10.1371/journal.pone.0242193 (PMC7671561; doi:10.1371/journal.pone.0242193)
Supplement: S3 File — (DOCX) [file pone.0242193.s003.docx]

**S3 File. Invitation letter and consent form.**

**INVITATION LETTER – Inuit knowledge about Arctic tern distribution, abundance and habitat in Nunavik**

Dear______________________________

You are invited to participate in the research project *Inuit knowledge about Arctic tern distribution, abundance and habitat in Nunavik*. The project is being conducted and funded by Environment and Climate Change Canada and the Nunavik Marine Region Wildlife Board, in collaboration with Acadia University and the Arctic Eider Society. This research is also supported by the Regional Nunavimmi Umajulivijiit Katujaqatigininga (RNUK). The objective of this project is to document Inuit knowledge about Arctic tern distribution, abundance and habitat in Nunavik in order to support ongoing conservation initiatives and identify community-based monitoring opportunities for this species in Nunavik.

**Procedures**

To fully participate in an interview, you will need to provide approximately ___ hour(s) of your time. The discussion will be audio recorded, photographed, and notes will be taken. We will be meeting at ________*(place)*__________ on _______*(date)*_________ at _____*(time)*______. You will offered the amount of $______ for your participation in the interview. Light refreshments and snacks will be provided.

The information and opinions that you share during the interview will be included in the results of this project and will be shared publicly in the form of reports, publications, or related project outputs (e.g., maps, posters, presentations, news items, postings on the internet). With your permission, original audio, photo, transcript, and/or map recordings will be stored and publicly accessible for future use in this community for heritage or education purposes, and/or for future research projects conducted by project team members.

**Contact information**

If you have any questions or concerns about this project, or the consent you have provided, please contact Frankie Jean-Gagnon, Dominique Henri or your Local Nunavimmi Umajulivijiit Katujaqatigininga (LNUK).

Frankie Jean-Gagnon Dominique Henri

*Lead Researcher Project Leader*

Wildlife Biologist, Nunavik Marine Region Indigenous Knowledge Specialist

Wildlife Board Environment and Climate Change Canada

P.O. Box 433 105, McGill Street, 7^th^ Floor

Inukjuak, QC, J0M 1M0 Montréal, QC, H2Y 2E7

(819) 254-8667 (514) 496-9024

[fjeangagnon@nmrwb.ca](mailto:fjeangagnon@nmrwb.ca) [dominique.henri@canada.ca](mailto:dominique.henri@canada.ca)

**CONSENT FORM – Inuit knowledge about Arctic Tern distribution, abundance and habitat in Nunavik**

I have received the invitation/information letter that goes along with this consent form. I have been fully informed of the objectives of this project. I understand these objectives and consent to being interviewed for the project. I understand the interview will be audio recorded, and that photographs will be taken with my permission. I understand that steps will be undertaken to ensure that this interview will remain confidential unless I consent to being identified. I understand that the information I share during the interview will be used by the research team in reports and publications related to this project. I also understand that, if I wish to withdraw from the study, I may do so without any repercussions.

I would like my name and the information I provide used as follows (please tick):

| I want my name included in a list of participants who contributed to the interviews.  I DO NOT want my name included in a list of participants who contributed to the interviews. |
| --- |
| I want to provide a brief personal biography to the research team and I give permission for it to be included in any reports and publications related to this project.  I DO NOT want to provide a brief personal biography to the research team. |
| I want to provide a photograph of myself to the research team and I give permission for it to be included in any reports and publications related to this project.  I DO NOT want to provide a photograph of myself to the research team. |
| I want my original audio, photo, transcript, and/or map recordings to be publicly accessible and stored for future use in this community and on SIKU for heritage, education or research purposes.  I DO NOT want my original audio, photo, transcript, and/or map recordings to be publicly accessible. |
| **Participant consent: _______________________________________________________**_ (print name)    **________________________________________________________** (sign name)  **Verbal consent, check here:**  **Date(s) of consent:** __________________________________________________________  **Witness signature:** __________________________________________________________ |
