# Supplementary material for: Inuit knowledge of Arctic Terns (Sterna paradisaea) and perspectives on declining abundance in southeastern Hudson Bay, Canada
Source: PLoS One. 2020 Nov 17;15(11):e0242193. doi: 10.1371/journal.pone.0242193 (PMC7671561; doi:10.1371/journal.pone.0242193)
Supplement: S4 File — (DOCX) [file pone.0242193.s004.docx]

**S4 File. Final results and data storage**

We presented final project results in person to members of the Nunavik Marine Region Wildlife Board (December 2019) and Kuujjuaraapik residents (January 2020). We prepared a final project report (available in English and Inuttitut) for the community of Kuujjuaraapik and project partners (Henri et al., 2019). With permission from study contributors and the LNUK of Kuujjuaraapik, all project data and results were stored at the LNUK of Kuujjuaraapik, the Nunavik Marine Region Wildlife Board and Environment and Climate Change Canada; project publications were archived on SIKU (a living online archive of Inuit knowledge and social mapping platform for northern communities) through collaboration with the Arctic Eider Society (see <https://siku.org/>). In March 2020, project partners finalized a data sharing agreement outlining data ownership, control, access, and protection measures (First Nation Information Governance Centre, 2014).

**References**

Henri, D., Jean-Gagnon, F., Weetaltuk, S., Mallory, M., Gilchrist, G., 2019. Nunavik Inuit Knowledge of Arctic terns: report to the community of Kuujjuaraapik and project partners. Unpublished report prepared for the Local Nunavimmi Umajulivijiit Katujaqatigininga of Kuujjuaraapik, the Regional Nunavimmi Umajulivijiit Katujaqatigi.

First-Nation-Information-Governance-Centre, 2014. Ownership, control, access and possession (OCAPTM): the path to First Nations information governance. Ottawa.
