# Supplementary material for: Inuit knowledge of Arctic Terns (Sterna paradisaea) and perspectives on declining abundance in southeastern Hudson Bay, Canada
Source: PLoS One. 2020 Nov 17;15(11):e0242193. doi: 10.1371/journal.pone.0242193 (PMC7671561; doi:10.1371/journal.pone.0242193)
Supplement: S5 File — (DOCX) [file pone.0242193.s005.docx]

### **S5 File. Additional discussion on Arctic Tern ecology**

**Additional discussion**

### **Migration**

Arctic Terns spring return from Antarctica occurs in April and May (Egevang et al., 2010). Around the community of Kuujjuaraapik (55°N), Nunavik, interviewees reported that Arctic Terns arrive progressively in small groups at the end of June and at beginning of July. Slightly different arrival periods have been observed for the rest of the Canadian Arctic and Greenland. In the High Canadian Arctic, on Nasaruvaalik Island (75°N) in Penny Strait, Nunavut, Arctic Terns start to arrive early June with a peak at the end of the month (Mallory et al., 2017). Similarly, ten terns fitted with miniature geolocators migrated back to the High Arctic Sand Island (74°N) in northeast Greenland in late May and early June of 2007 (Egevang et al., 2010). Although for this same location, another study revealed that Arctic Terns arrive to breed between late June and early July (Egevang and Frederiksen, 2011). Interannual variations in the timing of tern arrival to their breeding grounds is consistent with testimonies from Kuujjuaraapik contributors, who explained that this timing can vary according to environmental conditions, such as temperature and presence of snow or ice (as previously reported in Drury, 1960). Interviewees also explained that Arctic Terns return to the same area to breed every year, although they may choose different islands to nest if they encounter predators. These observations are similar to what has been reported by biologists to date. Indeed, Arctic Terns generally display high fidelity to breeding colonies (Devlin et al., 2008), particularly at a regional level (Egevang, 2010). Arctic Terns can also move between neighbouring breeding sites across years in response to local disturbance such as predation or food availability (Egevang and Frederiksen, 2011; Egevang, 2010; Brindley et al., 1999). Furthermore, they can nest in different areas as places become free of snow and available for nest sites, as observed on Bear Island in the Norwegian Arctic (Lack, 1933).

In the Kuujjuaraapik region, Inuit harvesters reported that Arctic Tern fall migration occurs during the first two weeks of September, generally before the first snowfall, when temperatures decrease. Similarly, Arctic Terns probably departed from Nasaruvaalik Island between mid to late September, just after researchers had left the island (Mallory et al., 2017). For the 11 Arctic Terns tracked from breeding colonies in Greenland and Iceland, fall post-breeding migration occurred between August and November (Egevang et al., 2010). These terns then took two southbound migration routes in the South Atlantic, one following the west African coast and the other the Brazilian coast, to reach the Antarctic ocean where they stayed between December and March (Egevang et al., 2010).

**Reproduction**

Around Kuujjuaraapik, contributors reported that Arctic Terns lay their eggs in late June and early July; terns start laying when the weather is warm and the ice and snow have melted. The absence of ice keeps away terrestrial predators from nesting islands, and gives birds access to open water to feed. Similar egg laying periods were reported across the Arctic, although variations likely exist depending on annual sea ice conditions. For example, in Kitsissunnguit, in western Greenland, egg laying usually takes place mid-June, but some years it took place three weeks earlier as sea-ice retreated earlier (Egevang, 2010). In the Canadian High Arctic, on Nasaruvaalik Island in Nunavut, the first Arctic Tern eggs were observed by biologists at the end of June, with an overall mean at the end of the first week of July, according to observations made between 2007 and 2011 (Mallory et al., 2017). In 1954, on Bylot Island (73°N) in Nunavut, the first eggs were found during the second week of July (Drury, 1960), probably corresponding to a year when ice retreated late. Generally, among High Arctic marine birds, a key period for nesting corresponds to the last week of June and the ﬁrst week of July (Mallory et al., 2017).

Inuit harvesters from Kuujjuaraapik reported that Arctic Terns nesting around Kuujjuaraapik, generally lay two to three eggs per clutch. These numbers are similar to middle Europe clutch sizes as reviewed by Drury in 1960, but different from those observed in the High Arctic where Arctic Terns commonly lay between one and two eggs (Mallory et al., 2017; Egevang, 2010; Drury, 1960). One hypothesis that could explain this difference is that investment in a 3-egg clutch only occurs regularly at lower latitudes in the Arctic region, likely lower than 70° N (Egevang, 2010), as is the case for the Kuujjuaraapik area (situated around 55°N). Interestingly, according to Kuujjuaraapik contributors, Arctic Terns are able to lay new eggs to replace the ones that were collected or predated. A control experiment conducted in Greenland demonstrated that Arctic Terns are able to produce replacement clutches with minimum effect on the overall productivity, as long as eggs are removed early in season (Egevang, 2010).

In the Kuujjuaraapik region, Arctic Terns mostly lay their eggs on small islands located in coastal saltwater areas, although they can nest on islands located inland in freshwater lakes. Inuit harvesters also explained that these seabirds nest on flat grounds, generally in areas covered with small pebbles or gravel, but also sand, short grass, moss, small driftwood, as previously reported (BirdLife-International, 2018; Mallory et al., 2017; Gaston et al., 2012; Drury, 1960). According to contributors, Arctic Terns can also nest on big flat rocks, which has not been reported elsewhere.

Kuujjuaraapik residents had observed terns cohabiting peacefully on nesting islands with other bird species, including Black Guillemot (*Cepphus grille*), Long-tailed Duck (*Clangula hyemalis*; also known as Oldsquaw), Canada Goose (*Branta canadensis*), eiders and gulls – though gulls can depredate tern eggs. Similarly, in the Canadian High Arctic, breeding associations were reported between Arctic Tern and Common Eider (*Somateria mollissima*), Long-tailed Duck, Sabine’s Gull (*Xema sabini*), Black-legged Kittiwake (*Rissa tridactyla*) and Thick-billed Murre (*Uria lomvia*) (Mallory et al., 2017, 2010; Pratte et al., 2016). In Greenland, breeding associations exist between colonies of Artic Terns and Sabine’s Gull, Ross’ Gull (*Rhodostethia rosea*), Red-necked Phalarope (*Phalaropus lobatus*) and Red Phalarope (*Phalaropus fulicarius*), as reviewed by Egevang (2010). It is possible that by nesting in close proximity to Arctic Terns, these birds exploit terns’ aggressive nesting behavior to minimize risk of nest predation. However, a study conducted in the Canadian High Arctic revealed that Common Eider nests located within Arctic Tern colonies were actually more predated, suggesting that nesting in association with Arctic Terns offers no obvious beneﬁts to Common Eiders (Pratte et al., 2016).

In the Kuujjuaraapik area, Inuit harvesters reported that chicks hatch around three weeks after eggs were laid, as previously reported in the High Arctic). Indeed, Arctic Terns spent around 21 days incubating, although this can differ between years (Mallory et al., 2017). As Kuujjuaraapik contributors reported that the first eggs were laid at the end of June, the fist chicks should appear around middle July. However, when asked to comment specifically on tern hatching period, some contributors reported that hatching generally takes place in the first week of August, as reported for Arctic Terns on Nasaruvaalik Island in Nunavut in the High Canadian Arctic (Mallory et al., 2017). In Greenland, the majority of eggs hatched during the first week of July (Egevang, 2010).

### **Preys and predators**

Around Kuujjuaraapik, contributors observed that Artic Terns feed mostly on capelin (*Mallotus villosus*). Similarly, capelin was reported by scientists to be their most important prey in Kitsissunnguit, western Greenland (Egevang, 2010). Capelin is likely to be the key prey species for Arctic Terns across most of the northwestern Atlantic, including western Greenland, where capelin acts as a link between zooplankton and large vertebrates (Buren et al., 2014; Egevang, 2010; Carscadden et al., 2002). Kuujjuaraapik residents reported that Arctic Terns can also feed on sandlance (*Ammodytes spp.*), which was reported to be the main prey of Arctic Terns around islands from northern Scotland (Brindley et al., 1999). According to Kuujjuaraapik harvesters, sandlance is new to Hudson Bay. Actually, capelin and Atlantic sandlance (*Ammodytes americanus*) have spread to Hudson Bay, displacing Arctic cod (*Boreogadus saida*) in the diet of seabirds (Gaston et al., 2012). Inuit harvesters reported that invertebrates such as *Gammarus spp.* and mussels are also occasional preys of Arctic Terns around Kuujjuaraapik. Similarly, shrimps appeared in some years in the diet of Artic Terns at Kitsissunnguit, western Greenland (Egevang, 2010). At Kitsissunnguit, Arctic Tern chicks were also observed being fed with Arctic cod, other marine species (e.g., *Gammarus spp.*, *Pandalus spp.*), and terrestrial species (e.g., *Lepidoptera spp.*, arachnids) (Egevang, 2010). At Sand Island in northeast Greenland, Arctic Terns’ most important prey species was Arctic cod, with crustaceans (especially *Thysanoessa spp.*) as secondary prey items (Egevang, 2010). Throughout its breeding distribution, Arctic Terns feed on a high diversity of preys (i.e., fish, crustaceans, molluscs, insects, earthworms), with small fish being their most important food item (BirdLife-International, 2018; Egevang, 2010). Around Kuujjuaraapik, Inuit harvesters reported seeing Arctic Terns feed in association with seals. In Alaska, Arctic Terns were reported by Yup’ik hunters to feed in association with belugas (*Delphinapterus leucas*) (Fienup-Riordan, 1990), and, in Antarctica, biologists observed terns feeding in association with Antarctic minke whale (*Balaenoptera bonaerensis*) (BirdLife-International, 2018).

In the Kuujjuaraapik region, Inuit contributors reported that Arctic Tern predators were humans, Arctic foxes (*Vulpes lagopus*), polar bears (*Ursus maritimus*), wolves (*Canis lupus*), Glaucous Gulls (*Larus hyperboreus*), Herring Gulls (*Larus argentatus*), jaegers (*Stercorarius spp.*), Common Ravens (*Corvus corax*), Peregrine Falcons (*Falco peregrinus*) and Merlin (*Falco columbarius*). Similar species (i.e., polar bear, Arctic fox, Common Raven, Glaucous Gull, Long-tailed Jaeger [*Stercorarius longicaudus*], Parasitic Jaeger [*Stercorarius parasitica*], Pomarine Jaeger [*Stercorarius pomarinus*], Peregrine Falcon and Gyrfalcon [*Falco rusticolus*]) were observed to predate on Arctic Terns on Nasaruvaalik Island, Nunavut (Mallory et al., 2017). Common Raven, Parasitic Jaeger, Glaucous Gull and Great Black-backed Gull (*Larus marinus*) were also observed predating on Arctic Tern eggs and chicks at Kitsissunnguit in Greenland (Egevang, 2010). In addition, Inuit contributors reported that Common Ravens have only recently began to feed on tern eggs in the Kuujjuaraapik region. Over the past decade, biologists have also commonly observed ravens predating on Arctic Terns in the High Arctic (Egevang, 2010; Mallory et al., 2017). Kuujjuaraapik residents affirmed that the presence of predators can prevent terns from laying eggs. Levermann and Tøttrup (2007) similarly suggested that Arctic Terns postpone egg laying in the presence of predators such as Arctic foxes or lay a replacement clutch if the first clutch has been predated (Egevang, 2010; Levermann and Tøttrup, 2007).

**References**

BirdLife-International, 2018. Sterna paradisaea, Arctic Tern. IUCN Red List Threat. Species. https://doi.org/http://dx.doi.org/10.2305/IUCN.UK.2018- 2.RLTS.T22694629A132065195.en

Brindley, E., Mudge, G., Dymond, N., Lodge, C., Ribbands, B., Steele, D., Ellis, P., Meek, E., Suddaby, D., Ratcliffe, N., 1999. The status of Arctic terns Sterna paradisaea at Shetland and Orkney in 1994. Atl. Seabirds 1, 135–143.

Buren, A.D., Koen-Alonso, M., Pepin, P., Mowbray, F., Nakashima, B., Stenson, G., Ollerhead, N., Montevecchi, W.A., 2014. Bottom-up regulation of capelin, a keystone forage species. PLoS One 9, 1–11. https://doi.org/10.1371/journal.pone.0087589

Carscadden, J.E., Montevecchi, W.A., Davoren, G.K., Nakashima, B.S., 2002. Trophic relationships among capelin (Mallotus villosus) and seabirds in a changing ecosystem. ICES J. Mar. Sci. 59, 1027–1033. https://doi.org/10.1006/jmsc.2002.1235

Devlin, C.M., Diamond, A.W., Kress, S.W., Hall, S.C., Welch, L., 2008. Breeding dispersal and survival of Arctic terns (Sterna paradisaea) nesting in the Gulf of Maine. auk 125, 850–858. https://doi.org/10.1525/auk.2008.07060

Drury, W.H., 1960. Breeding activities of long tailed jaeger, herring gull and arctic tern on bylot island, northwest territories, Canada. Bird-banding 63–79.

Egevang, C., 2010. Migration and breeding biology of Arctic terns in Greenland. Greenland In- stitute of Natural Resources, Dep. of Arctic Environment, NERI, Aarhus University & Department of Biology, Center for Macroecology, Evolution and Climate, University of Copenhagen. Greenland Institute of Natural Resources & National Environm.

Egevang, C., Frederiksen, M., 2011. Fluctuating Breeding of Arctic Terns (Sterna paradisaea) in Arctic and High-Arctic Colonies in Greenland. Waterbirds 34, 107–111. https://doi.org/10.1675/063.034.0114

Egevang, C., Stenhouse, I.J., Phillips, R.A., Petersen, A., Fox, J.W., Silk, J.R.D., 2010. Tracking of Arctic terns Sterna paradisaea reveals longest animal migration. Proc. Natl. Acad. Sci. U. S. A. 107, 2078–2081. https://doi.org/10.1073/pnas.0909493107

Fienup-Riordan, A., 1990. The bird and the bladder: the cosmology of central Yup’ik seal hunting. Études/Inuit/Studies 14, 23–38.

Gaston, A.J., Mallory, M.L., Gilchrist, H.G., 2012. Populations and trends of Canadian Arctic seabirds. Polar Biol. 35, 1221–1232. https://doi.org/10.1007/s00300-012-1168-5

Lack, D., 1933. 12. Nesting Conditions as a Factor controlling Breeding Time in Birds. Proc. Zool. Soc. London 103, 231–238. https://doi.org/10.1111/j.1096-3642.1933.tb01590.x

Levermann, N., Tøttrup, A., 2007. Predator Effect and Behavioral Patterns in Arctic Terns (Sterna paradisaea) and Sabine’s Gulls (Xema sabini) During a Failed Breeding Year. Waterbirds 30, 417–420. https://doi.org/10.1675/1524-4695(2007)030

Mallory, M.L., Boadway, K.A., Boadway, J.J.T., Akearok, J.A., 2010. Breeding Arctic terns kill lemmings. Arctic 63, 359–361. https://doi.org/10.14430/arctic1499

Mallory, M.L., Boadway, K.A., Davis, S.E., Maftei, M., Diamond, A.W., 2017. Breeding biology of Arctic terns (Sterna paradisaea) in the Canadian High Arctic. Polar Biol. 40, 1515–1525. https://doi.org/10.1007/s00300-016-2072-1

Pratte, I., Davis, S.E., Maftei, M., Mallory, M.L., 2016. Aggressive neighbors and dense nesting: nest site choice and success in high-Arctic common eiders. Polar Biol. 39, 1597–1604. https://doi.org/10.1007/s00300-015-1884-8
